# The influence of the nitrogen-to-protein conversion factors on the prediction of crude protein in black soldier fly larvae (*Hermetia illucens*) using near-infrared spectroscopy

**DOI:** 10.1007/s00216-025-06311-2

**Published:** 2026-01-08

**Authors:** S. Alagappan, J. R. Nastasi, L. C Hoffman, D. Cozzolino

**Affiliations:** 1 Centre for Nutrition and Food Sciences, Queensland Alliance for Agriculture and Food Innovation (QAAFI), The University of Queensland, Brisbane, QLD 4072 Australia; 2 School of Agriculture and Food Sustainability, The University of Queensland, Brisbane, QLD 4072 Australia

**Keywords:** Nitrogen, Black soldier fly, Near infrared, Nitrogen to protein factor

## Abstract

Protein determination is of importance to characterise insect composition and quality. While different factors (kp) to convert nitrogen to protein have been proposed to report the concentration of crude protein (CP) in insect applications, the N × 6.25 is still utilised by many commercial laboratories. Different data-dependent conversion factors have been proposed to report the concentration of CP in different insect species, including black soldier fly larvae (BSFL). The objective of this paper was to evaluate the effect of using different nitrogen to protein conversion factors (kp) to predict CP in BSFL from different commercial sources using near-infrared spectroscopy. The coefficient of determination in cross-validation (*R*^2^_CV_) and the standard error in cross-validation (SECV) for the prediction of CP% in the BSFL were 0.75 (SECV, 4.51%), 0.75 (SECV, 4.03%), and 0.75 (SECV, 3.43%), using the kp_6.25, kp_5.65, and kp_4.76, respectively. The study showed that the different kp used can affect the cross-validation statistics (SECV) for the prediction of CP in BSFL using NIR spectroscopy. Additionally, the lower accuracies obtained for the prediction of CP are not only associated with the kp used to calculate the CP but also with the amount of chitin in the BSFL. Understanding the variables, such as reference data, that influence the calibration results using NIR spectroscopy is of importance to better provide consistent QC methods for the industry. The limitations of this study are the few numbers of samples used to develop the calibration models, although different waste streams and larvae stages were evaluated.

**Graphical Abstract:**

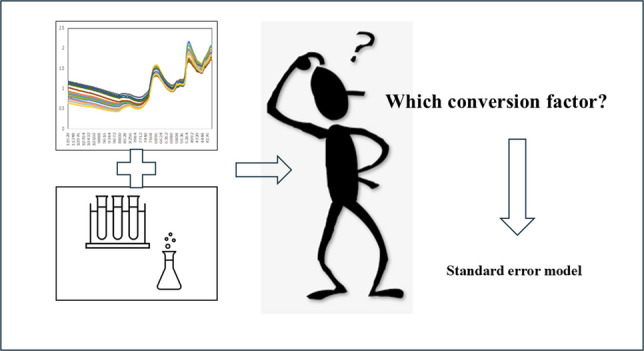

## Introduction

Sustainable sources of protein such as plant-based and insect proteins are of growing interest to feed the increasing demand for high value proteins to support the growing population [1–3]. Recently, it has been reported that insects (e.g. larvae, flour) can be considered a potential source of suitable protein for animals and humans [1–3]. The high value protein content of insects in the range of 40 − 75% on a dry matter (DM) basis, which makes insects a sustainable and inexpensive protein alternative for both human food and animal feed use [4–9]. The nutritional value of insects, in addition to the ease of rearing, makes this ingredient especially interesting to be used as an ingredient in food and feed production when the insects are in the larval stage [7–10]. Furthermore, the utilisation of insects as an alternative source of protein requires efficient and consistent methods to quantify protein content (e.g. Dumas vs. Kjeldahl, nitrogen to protein conversion factor) by the industry and authorities [11].

The routine analysis of crude protein (CP) utilises an overall nitrogen to protein conversion factor (kp) (N) × 6.25 [12]. This factor is applied to report the concentration of CP in most food and feed products, including insects [12–14]. This factor is derived from the early research on proteins of animal origin which contain approximately 16% nitrogen [12–14]. However, it has been reported that individual factors must be used depending on the feed or food types (e.g. species, origin) [12–14]. Furthermore, different regulations and standards have defined that N × 6.25 is the factor to be used to report the amount of CP in foods “except when the official AOAC procedure for a specific food requires another factor” [12–14]. For example, the AOAC methods described to determine protein content in cereals and cereal adjuncts specify a kp of N × 5.7 for wheat and sub-products and 6.25 for other cereals [12–14]. It has been reported that the kp of N × 5.7, used for the calculation of CP in wheat and wheat flour, is based on the nitrogen content of gliadin and glutenin, the predominant protein groups in wheat endosperm [12–14]. Research in CP methods highlighted that individual kps are required for different grains, their milled products or subproducts, where this variation can be the result of the constituent amino acid profile of the protein in the grains or subproducts [12–14].

In the same way, the determination of CP content of insects has been reported by different authors, where it can range between 37 and 63% of DM [11, 15, 16]. This variation in chemical composition (e.g. protein content) is due to a variety of production and post-harvest variables including diet quality and quantity, larvae density, and physiological stages (e.g. instars) [11, 15, 16]. Another important consideration is that, in addition to protein and fat content, insects contain around 2 up to 10% of chitin, a modified polysaccharide that contains nitrogen [11, 15–18]. The reported amount of CP of different insect species varies due to the different nitrogen to protein kp used where the Nx6.25 is generally used [11, 15–19]. However, rather than 6.25 (the default used by commercial laboratories), other conversion factors have been used; for example, 5.62 suggested for extracts of BSFL and using 4.76 whole larvae [11, 15–19].

The presence of nonprotein nitrogen (NPN) in insects, such as chitin, nucleic acids, phospholipids, and excretion products (e.g. ammonia) in the intestinal tract, also contributes to an overestimation of the protein content [15, 20]. Different authors have reported that the amount of nitrogen from chitin would not significantly increase the total amount of nitrogen [19–21].

Knowing the protein profile, the protein functionality, and consequently its quality can be an important strategy to increase consumer’s acceptance of insect ingredients used in food formulation development. The utilisation of black soldier fly larvae (BSFL) as a source of high-value protein has increased [7–9, 22–25]. The BSFL is also rich in saturated fatty acids, in particular, lauric acid (C12:0), where a large amount of structural and muscular proteins has been identified in BSFL [7–9, 22–25]. This has prompted the utilisation of insect protein as an alternative source to replace others such as meat and soybean [7–9, 22–25]. Moreover, antimicrobial peptides and some enzymes, such as trypsin and chymotrypsin, were identified, which could be, in the near future, of industrial interest [7–9, 21–25]. Therefore, the utilisation of consistent and efficient analytical methods is desirable for use by industry, feed mills, or regulation authorities to guarantee its quality [7–9, 21–25]. For this purpose, different nitrogen to protein conversion factors were evaluated and reported by different authors to estimate CP in BSFL (e.g. 5.62 for protein extract, 4.67 for larvae flour) [11, 17–19].

More recently, new techniques such as near-infrared (NIR) spectroscopy have been evaluated and implemented to predict the proximate composition (e.g. protein, fat content) in different insect’s species including BSFL [26–30]. However, the accuracy of the calibration models developed using NIR spectroscopy can be affected by different variables such as particle size, sampling, and reference error [31, 32]. Therefore, knowing these variables will help to better understand the statistics obtained [31, 32].

The objective of this paper was to evaluate the effect of using different nitrogen to protein conversion factors to estimate crude protein in black soldier fly larvae analysed using near-infrared spectroscopy.

## Materials and methods

Two sample populations of BSFL were utilised in this study, BSLF SW cultivated on a soy waste diet obtained from a commercial custard manufacturer and BSLF BV cultured on a customised bread–vegetable diet. Details about the rearing and post-harvest conditions and feed sources can be found elsewhere [29, 30]. The homogenised larvae samples were then analysed using both NIR spectroscopy and proximate analysis. A Bruker Tango-R spectrophotometer (Bruker Optics GmbH, Ettlingen, Germany) with a gold-coated integrating sphere (diffuse reflection) was used to collect the NIR spectra of the samples. Samples were placed in a borosilicate glass cuvette with a diameter of 10 mm. The spectral data was collected in reflectance and recorded using OPUS software (version 8.5, Bruker Optics GmbH, Ettlingen, Germany) with 64 interferograms at a resolution of 4 cm^−1^ in the wavenumber range of 11,550 to 3950 cm^−1^ [29, 30]. Between samples, the borosilicate cuvettes were cleaned with 70% water/ethanol (v/v) solution and wiped using paper towels between samples.

The content of crude protein in the BSFL samples was carried out by the analytical services unit of the School of Agriculture and Food Science, The University of Queensland (St Lucia, QLD, Australia). The analytical laboratory followed the AOAC standard protocols (AOAC method 992.15) to determine CP (total nitrogen × 5.62). The amount of nitrogen provided by the commercial laboratory was used to calculate the different concentrations of CP based on the different nitrogen to protein conversion factors (kp), namely 6.25, 5.62, and 4.76 [11].

The Unscrambler software (version X, Camo, Oslo, Norway) was used to develop the chemometric models. Before any further analysis, the NIR spectral data were smoothed and pre-processed using the Savitzky–Golay second derivative (second-order polynomial and a smoothing window size of 10 points) [33]. In this study, partial least squares (PLS) regression was used to develop the calibration models for CP using three kp factors. The models were developed using leave-one-out cross-validation (full cross-validation) [34, 35]. The statistics calculated and used to evaluate the PLS models included the coefficient of determination in cross-validation (*R*^2^_CV_), slope, bias, the number of latent variables (LV), the standard error in cross-validation (SECV), and the ratio of performance deviation (RPD) values. The RPD values were calculated as the relationship between the standard deviation in composition measured using the reference method divided by the SECV [34, 35].

## Results and discussion

Table 1 shows the descriptive statistics (average, range, and standard deviation) for the concentration of CP % calculated using three kp evaluated in this study. The average CP% varies between 35.5% calculated using the kp_4.76 and 46.7% calculated using the kp_6.25. The coefficient of variation did not differ between the different factors used to calculate the CP%. Overall, the range in CP% was considered adequate and used to develop the NIR calibration models.

**Table 1:** Descriptive statistics (average, range, and standard deviation) for the concentration of crude protein (%) in black soldier fly larvae calculated by applying three nitrogen to protein conversion factor (kp) and used to develop the near-infrared calibrations

	kp_6.25	kp_5.62	kp_4.76
Average (%)	46.7	41.9	35.5
SD	8.65	7.78	6.57
Range	27.6–62.0	24.8–55.7	21.0–47.0
CV%	18.5	18.5	18.5

*CV* coefficient of variation (SD/average × 100), *SD* standard deviation

Table 2 shows the cross-validation statistics for the prediction of CP using three nitrogen to protein conversion factors as detailed in the materials and methods section. The coefficient of determination in cross-validation (*R*^2^ cv) and the standard error in cross-validation for the prediction of CP in the BSFL larvae was 0.75 (SECV, 4.51%), 0.75 (SECV, 4.03%), and 0.75 (SECV, 3.43%), using the kp_6.25, kp_5.65, and kp_4.76, respectively. Recently, Smets and collaborators [11] have reported a degree of variation in the kp factors used to calculate CP% in BSFL. The authors highlight that the CP% varied depending on the rearing substrate utilised and the larval instars sampled [11]. Their work also noted that regardless of any biological variation, a kp factor of 4.43 provided a more accurate prediction of CP than the standard kp of 6.25 [11]. These authors also highlighted that despite this biological variation observed, a kp factor of 4.43 will be more accurate to predict CP compared to the kp of 6.25 [11]. The results of this study indicated that the SECV values obtained using the kp 4.76 were the lowest and agreed with those reported by other authors [11]. The SECV was also analysed by means of a statistical analysis. Statistical significant differences were observed when the SECV using kp_6.25 was compared with the SECV using kp_4.76. No statistically significant differences were observed for the other kp used to calculate the concentration of CP.

**Table 2:** Cross-validation statistics for the prediction of crude protein in black soldier fly larvae using three different conversion factors and analysed using near infrared spectroscopy

	kp_6.25	kp_5.62	kp_4.76
*R*^2^_CV_	0.75	0.75	0.75
SECV	4.51	4.03	3.43
Bias	−0.04	−0.03	−0.06
Slope	0.74	0.74	0.74
LV	4	4	4
RPD	2	2	2

*kp* nitrogen protein factor, *R*^*2*^_*CV*_ coefficient of determination in cross-validation, *SECV* standard error in cross-validation, *LV*: latent variables, *RPD* residual predictive deviation (SD/SECV)

In addition to the *R*^2^_CV_ and SECV, the RPD values obtained were equal to 2 for all the models developed. This value indicated that the cross-validation results are only good for the semi quantitative analysis of the samples [34, 35]. The lower cross-validation statistics obtained for the prediction can also be explained by the narrow range in CP as reported in the literature [27, 29, 30].

**Fig. 1:**
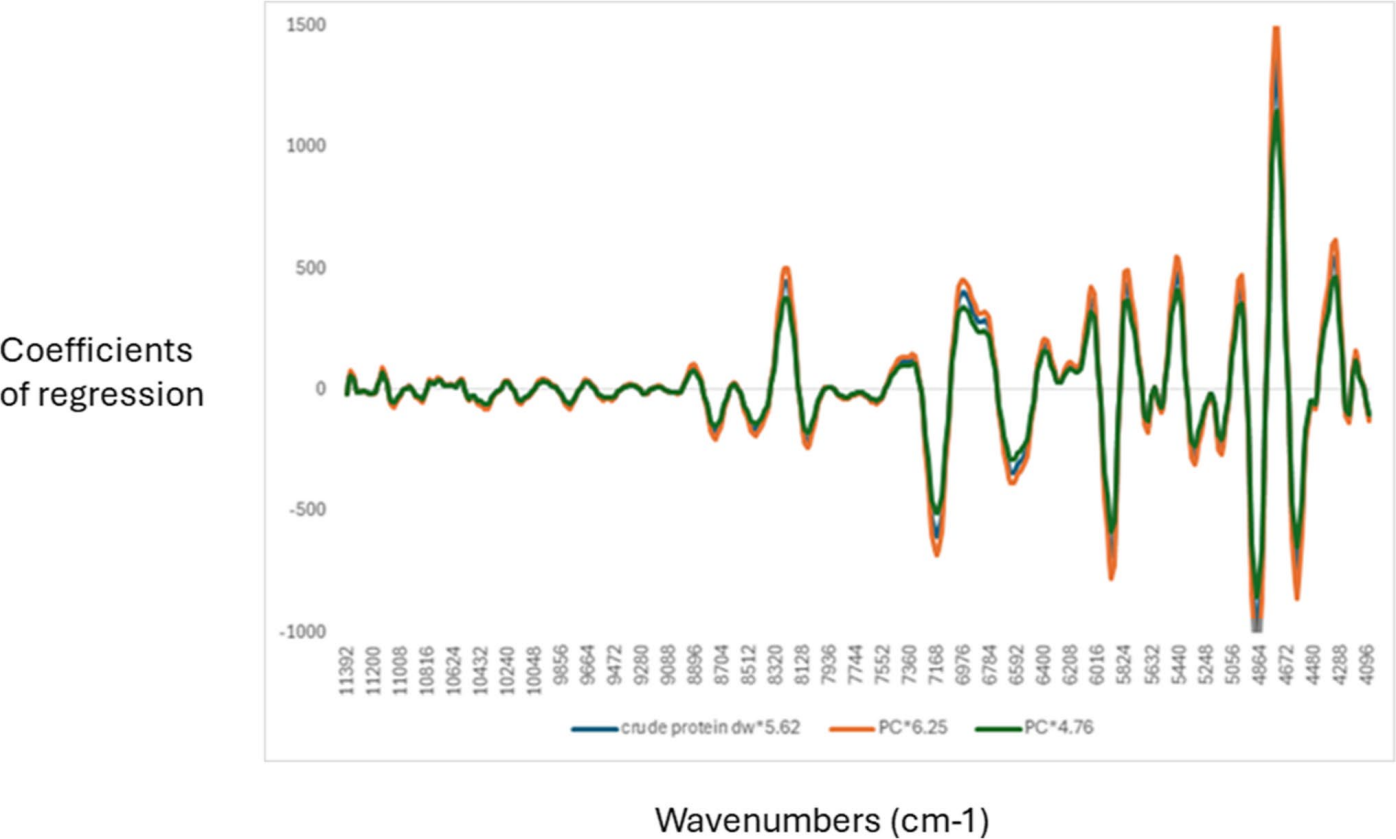
Coefficients of regression used by the partial least squares regression models used to predict crude protein in black soldier fly larvae using three different nitrogen to protein conversion factors

The coefficient of regression used by the PLS models was evaluated and interpreted, as they indicate the main wavenumbers used by the model. Figure 1 shows the coefficients of regression for the three cross-validation models developed using the kp. The highest coefficient of regression was found in wavenumbers around 8752 cm^−1^ associated with C-H stretching bonds derived from aromatic groups, around 8462 cm^−1^ associated with both C-H stretching bonds derived from aryl groups, and C-H (2υ) stretching vibration of -CH_2_, while around 8096 cm^−1^ this band is associated with C = O (3υ) stretching bonds from amide groups, symmetric N–H + amide II in the combination bands related to protein groups [30, 36–39]. Around 5920 cm^−1^ associated with C-H aromatic groups, around 5664 cm^−1^ associated with C-H_2_ bonds, around 5328 cm^−1^ and 5152 cm^−1^ associated with O–H, mainly water content. Around 4840 cm^−1^ this wavenumber is associated with both N–H bonds derived from amides and the combination of secondary amides of proteins, around 4592 cm^−1^ associated with both N–H and N–H combinations bonds [29, 35–38]. Slight differences were observed in wavenumbers around 7168 cm^−1^ (C-H_2_) and around 6624 cm^−1^ associated with both N–H amide and aromatic groups [30, 36–39]. The magnitude of the coefficients of regression used for the kp_4.73 models was slightly smaller compared with the coefficients derived from the models from the other two factors. It has also been reported that wavenumbers around 8368 cm^−1^, 6623 cm^−1^, and 4687 cm^−1^ might be associated with chitin [39]. These bands are associated with second overtone C-H stretching, first overtone N–H stretching, and N–H stretching plus amide II, respectively. These wavenumbers indicated the presence of chitin as they might add N–H into the analysis [30, 36–40]. In this study, wavenumbers around 6623 and 4687 cm^−1^ were observed where one of these wavenumbers corresponds to the absorption of chitin in agreement with published results by other authors [30, 36–40].

Insects can have a high concentration of chitin depending upon their developmental stage and other extrinsic factors, as this compound is one of the main constituents of their exoskeleton [40]. It has been reported that in the case of BSFL, the concentration of chitin is higher in the 6th instar larvae as the larvae reach the adult stages [41, 42]. Other authors indicated that the prediction of CP using a combination of NIR spectroscopy with PLS regression might be influenced by the concentration and distribution of chitin in the insect samples analysed [30, 36–40]. This was best illustrated by similar cross-validation statistics studies of other insect species, including Tenebrio and crickets [28, 43–46]. The main limitation of this study is the limited number of samples used to develop the models, where the models must validate using an independent set of samples (samples not included in the calibration). The practical implications of these results are that the concentration of CP can be underestimated or overestimated by up to 1% DM. The use of this over-or-under estimated protein from BSFL in feed and other dietary formulations will not exhibit desired technical and functional characteristics and will need additional fine-tuning by the end user to meet their targeted quality. The results of this study also showed how the error or the use of an adequate nitrogen-to-protein conversion factor can influence the accuracy of the NIR models developed. As described by other authors, differences in nitrogen content in a wide range of foods and feed ingredients can be influenced by how and which nitrogen-to-protein factor is used to report the CP of foods and insects [16, 18–22]. Furthermore, the determination of CP in insects continues to be a challenge, as shown in this study. This has also influenced the statistics obtained after the application of rapid methods such as spectroscopy.

## Conclusions

The study showed how the different nitrogen to protein conversion factors used can affect the cross-validation statistics for the prediction of CP in BSFL using NIR spectroscopy. Additionally, the lower accuracies obtained for the prediction of CP are not only associated with the conversion factor used to calculate the CP but also with the amount of chitin in the BSFL. Understanding the variables, such as reference data, that influence the calibration results using NIR spectroscopy is of importance to better provide consistent QC methods for the industry. The limitations of this study are the few numbers of samples used to develop the calibration models, although different waste streams and larvae stages were used.

## Data Availability

Data will be made available on request.
